# Results of Total Hip Replacement Surgery Using Short-Stem Spiron Prosthesis in Vietnamese Adults

**DOI:** 10.1155/2024/4623071

**Published:** 2024-07-09

**Authors:** Mai Duc Thuan, Nguyen Quoc Dung

**Affiliations:** Department of Joint Surgery Institute of Trauma and Orthopedic – 108 Military Central Hospital, Hanoi, Vietnam

## Abstract

**Introduction:**

Total hip arthroplasty with traditional stem joints, although bringing many benefits to patients indicated for hip replacement, faces many difficulties in reoperations in young patients due to the limited lifespan of the artificial joint. Recently, the short-stem joint was introduced to help overcome this problem. This study aims to evaluate the results of short-stem implants for primary total hip arthroplasty in Vietnamese adults.

**Materials and Methods:**

The study design is retrospective longitudinal. The study subjects were 91 patients with aseptic necrosis of the femoral head who underwent short-shaft cementless total hip replacement surgery from February 2012 to December 2018. Outcomes that were assessed included implant survivorship, Harris hip scores, thigh pain, periprosthetic fracture, subsidence, proximal stress shielding, the prevalence of stem malalignment, and inappropriate implant sizing.

**Results:**

Our research group has 119 hips of 91 patients (28 patients had hip replacements on both sides), with the average follow-up time of 67.1 months. Seven cases of early joint loosening required replacing the traditional stem. The survival rate is 94.1%. The average Harris hip score 24 months after surgery was statistically significantly higher than before (92.00 ± 4.47 and 45.56 ± 3.22, respectively, *p* < 0.001). The average leg length discrepancy between the two limbs before surgery was 9.5 ±mm and 24 months after surgery was 3.4 ± 2.9 mm. There were 12 cases (10.1%) of partial bone loss around the femoral stem; 12 cases of joint displacement of more than 2 mm, accounting for 10.1%; and 1 case of acetabular fracture, accounting for 0.8%.

**Conclusion:**

The Spiron short stem has certain advantages suitable for young patients, restoring the hip anatomy and significantly improving postoperative hip function. However, this is a complex technique that needs to be performed by experienced surgeons who have practiced for a long time. We encourage using the short-stem implant during primary total hip arthroplasty in Vietnamese adult patients.

## 1. Introduction

Total hip arthroplasty for the aseptic necrosis of the femoral head has become popular worldwide, and its great effectiveness is undeniable [1–3]. Although aseptic necrosis usually occurs in the 40–60 age group, the number of young patients with this disease in Vietnam is increasing daily. Although there are many improvements in materials and surface coatings, the latest generations of joints only have an average lifespan of 15–25 years, so young patients who have had hip replacements will have to face second- and third-time joint replacements after their first joint replacement [4, 5].

Joint replacement for the second and third time involves a technique with great difficulty and complexity. It has a considerably higher rate of complications and symptoms than joint replacement for the first time. How can we reduce difficulties, lower complication rates, and improve treatment effectiveness for hip replacement patients? The current trend supported by many people is that when replacing a joint for the first time, it is necessary to save as much healthy bone as possible, creating favorable conditions for future joint replacements. Recently, people have used short-stem joint to replace traditional-stem joints when performing initial joint replacement to preserve bones in the neck and upper femoral head to overcome the abovementioned difficulties [6, 7]. We conducted this study intending to evaluate the results of short-stem implants for primary total hip arthroplasty in Vietnamese adults.

## 2. Materials and Methods

### 2.1. Study Settings

The study design is retrospective longitudinal. The study was conducted at the Institute of Trauma and Orthopaedics, 108 Vietnam Central Military Hospital.

**Figure 1:**
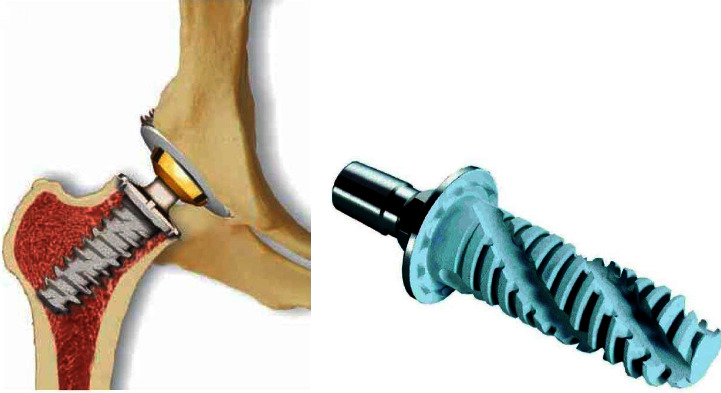
Image of Spiron total hip joint (a) and femoral stem joint (b).

**Figure 2:**
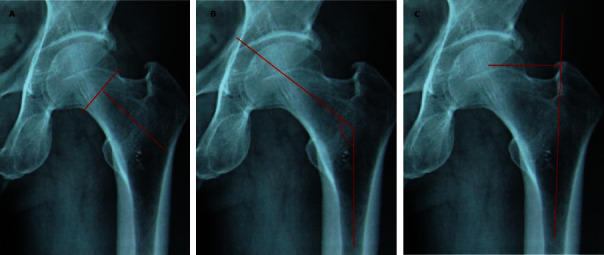
Indicators related to the selection and placement of the Spiron joint shaft. (A) Head base to the inner border of the outer wall of the femur, (B) femoral shaft neck angle, and (C) distance from the center of the head to the anatomical axis of the bone).

**Figure 3:**
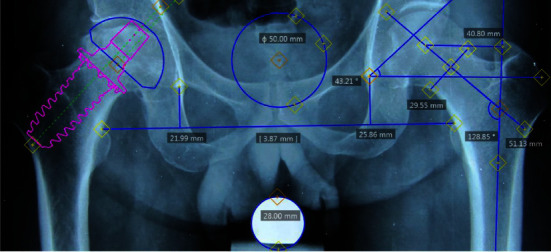
Preoperative planning on X-ray based on 2D software.

The study subjects were 91 patients with aseptic necrosis of the femoral head who underwent short-shaft cementless total hip replacement surgery from February 2012 to December 2018, including 28 patients with two sides replaced and 63 with one side replaced. Selection criteria are patients from 18 to 50 years old diagnosed with aseptic necrosis of the femoral head stages IV, V, and VI. According to Steinberg's classification, damage can be unilateral or bilateral. The shape and quality of the femoral neck bone are still good, and the femoral neck has not been deformed. On MRI, the femoral neck has no damage (no necrotic foci). The patient received total hip replacement surgery using a short-stem Spiron® prosthesis (Figure 1), which was planned preoperatively using 2D software (Figures 2 and 3). The part of the femoral neck that does not have necrotic foci is considered at the intended location of the femoral neck cut on the preoperative plan. Exclusion criteria are patients who cannot fully straighten their legs or cannot internally rotate their feet and patients who do not have follow-up appointments or have a follow-up period of fewer than 24 months.

### 2.2. Evaluated Results after Surgery

Time for the postoperative follow-up is as follows: immediately after surgery, 8–12 weeks after surgery, 12 months, and once a year. Outcomes that were assessed included implant survivorship, Harris hip scores, thigh pain, periprosthetic fracture, subsidence, proximal stress shielding, the prevalence of stem malalignment, and inappropriate implant sizing. We assessed hip function according to Harris [8].

### 2.3. Statistical Analysis

The data were analyzed using IBM SPSS Statistics for Windows, version 22.0. Categorical variables were presented as total numbers and percentages and continuous variables as means and standard deviations. An independent sample *t*-test tests for the difference between two groups when variable is normal. A Mann–Whitney *U* test determines the difference between the two groups when the variable is skewed. A *p* value of <0.05 was considered statistically significant, and all tests were two-tailed.

## 3. Results

### 3.1. Patient Characteristics

Our research group has 119 hips of 91 patients (28 patients had hip replacements on both sides). The average age is 41.22 ± 5.97 (25–50), and the group from 41 to 50 accounts for the most (57.1%). Men make up the majority, with 97.8%. We mainly encountered patients with stage IV and V lesions, of which stage V accounted for the most significant proportion with 50.4% (Table 1).

### 3.2. Characteristics of Implant

The metal-backed acetabular cup is the most used, with 59 joints accounting for 49.6%. 100% of the patients received caps with a diameter of 28 mm, mainly −5 mm femoral head with 79.8%. Joints with a diameter of 20 are used the most, with 73 joints accounting for 61.3%. The length of the femoral stem has the following four types: 45, 50, 55, and 60 mm. Joints with a length of 50 mm are used the most, with 53.8% (Table 2).

### 3.3. Far Results

**Figure 4:**
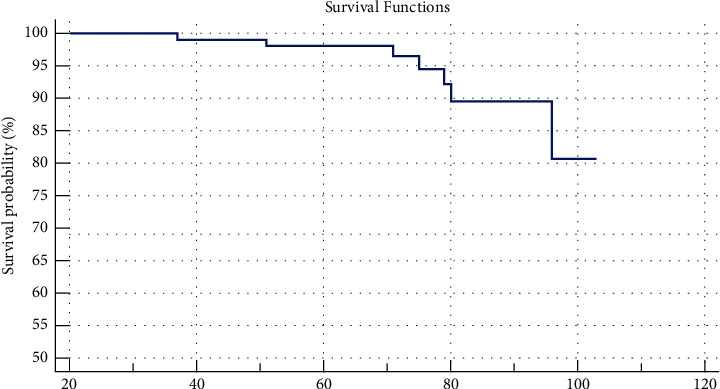
Kaplan–Meier survival curves with 95% confidence interval.

The shortest follow-up time is 24 months and the longest is 103 months. The average follow-up time is 67.1 ± 20.74 months. Seven cases of early joint loosening required replacing the traditional stem. The survival rate is 94.1% (Figure 4).

The average Harris hip score 24 months after surgery was statistically significantly higher than before (92.00 ± 4.47 and 45.56 ± 3.22, respectively, *p* < 0.001). There are 94/119 joints rated very good, accounting for 79%, 22/119 joints rated good, accounting for 18.5%, 3/119 joints rated average, accounting for 2.5%, and no patients rated poor (Table 3).

The average leg length discrepancy between the two limbs before surgery was 9.5 ± 2.3 (0.02–12.57) mm and 24 months after surgery was 3.4 ± 2.9 (0.02–8.00) mm; the difference is statistically significant.

### 3.4. Safety

There was 1 case of acetabular fracture, accounting for 0.8%. At the time of examination, after 12 months, the bone had completely healed, and the patient had no hip pain. Eleven cases (accounting for 9.2%) had persistent symptoms of discomfort in the greater trochanter area, but they were not frequent and did not reach the point of thigh pain.

There were 5 cases of external popliteal sciatic nerve damage, accounting for 5.5%. However, 2 cases of mild injuries recovered both movement and feeling within the first week. 2 patients recovered after one year. One case of paralysis did not fully recover, but there was still a sharp feeling in the soul.

There were 12 cases of partial bone loss around the femoral stem, accounting for 10.1%. Seventeen bright rims appeared around the artificial joint on X-rays, accounting for 14.3%, of which 12 cases of joint displacement of more than 2 mm accounted for 10.1%, all of which were angulation displacements.

## 4. Discussion

In recent years, the number of hip replacements for young patients has become increasingly popular [9]. In our clinical research group, patient ages ranged from 25 to 50 years, with an average of 41.6 years old. The problem is, why choose a short-stem hip joint for young patients? Currently, the lifespan of a traditional-stem hip joint ranges from 15 to 25 years, so patients under 50 years old are at high risk of having to have their hip replaced. Floerkemeier et al. believed that short-stem hip replacement to treat aseptic necrosis of the femoral head for young patients will help preserve bone ideally and have more options when replacing the joint. According to him, the benefit of preserving part or all of the femoral neck is to retain a “cushion” that can withstand physiological forces and is better than fixing the femoral head in a traditional-stem hip replacement [10]. This physiological force-bearing cushion will help reduce tension at the pressure site (reduce stress shielding phenomenon), thereby reducing the rate of aseptic lamination loosening. Also, according to the author, most short handles have a larger diameter of the upper end than the lower end; this design increases the stability of the joint when rotating and moving.

Retaining the femoral neck in hip replacement has existed since the 1980s. Freeman is mentioned as the father of maintaining the femoral neck in the classic article “Why restore the neck?” [11]. Stulberg and Patel stated that preserving the neck will be difficult with long-stem hip replacement [12]. In 1979, Pipino and Calderale were the first to retain the entire femoral neck when replacing the short hip joint. He was also the first to use the term “tissue paring” from which the short-stem joint is also known as the “bone-preserving joint” [13]. Over the past decade, the number of short-stem hip replacement surgeries has continued to increase. Short-stem hip replacement or surface hip replacement both makes it easier for the surgeon to replace it. However, most surgeons prefer short-shaft hip replacements for younger patients even though surface hip replacements provide more pronounced bone preservation of the proximal femur. Why is that? According to Lazarinis et al. and Johanson et al., surface hip replacement has a high rate of femoral neck fractures, early loosening of the shaft due to osteonecrosis, pseudotumours phenomenon, and many other complications that have decreased the popularity of surgeons [14, 15].

According to Birkenhauer B. and McTighe T., the fixed short-stem hip replacement technique in the femoral neck area has many differences compared to long femoral stem hip replacement [16, 17]. Because the medullary canal of the femoral neck is smaller and shorter than the medullary canal of the upper end of the femur, reaming and placing the short-stem joint at the femoral neck is easier and faster than the long-stem joint. However, because it retains the entire femoral neck, it often covers the acetabulum, making it difficult to expose, ream, and place the acetabulum [18–20]. Sometimes, trying to push the femoral neck out of the acetabulum for reaming stretches the large sciatic nerve, affects the placement of the acetabulum (position, acetabular tilt angle), or causes soft tissue damage. If there is too much pressure on the femoral neck, it can be partially or entirely broken. These things affect the surgical results more or less. This is one of the most considerable difficulties surgeons face when replacing the Spiron joint. To overcome this problem, we proactively drilled a nail before reaming the acetabulum to determine the center of the femoral neck measured. We estimated the length of the femoral stem that would be replaced to trim the remaining femoral neck. In addition, you can use a specialized zigzag acetabular reamer or drop a separate reamer to the bottom of the acetabulum first and then insert the femoral stem and ream. In addition, the surgeon must be an experienced, well-trained hip replacement surgeon, performing joint replacement at facilities with complete specialized equipment for this surgery.

Also, according to McTighe, because the short stem fixed at the femoral neck lacks growth down to the femur's upper end and a guiding tool, it is difficult to place the femoral stem accurately in the desired position [17].

Banerjee S. conducted a literature search using the electronic medical databases PubMed, CINAHL Plus, EMBASE, and SCOPUS to identify all articles reporting the outcomes of short femoral stems in total hip arthroplasty [21]. The studies included 2734 hips in 2277 patients (mean age, 59 years; range, 36–79 years) at a mean follow-up of 4 years. The average survival rate of the abovementioned short-stem joints is 99.5% (97.5%–100%). This rate was 99.6% (97.5%–100%) with a follow-up period of at least two years. The survival rate in our study was 94.1%, with a mean follow-up period of 67 months.

About far result, the average Harris hip score 24 months after surgery was statistically significantly higher than before (92.00 ± 4.47 and 45.56 ± 3.22, respectively, *p* < 0.001). 94/119 joints rated very good, accounting for 79%, 22/119 joints rated good, accounting for 18.5%, 3/119 joints rated average, accounting for 2.5%, and no patients rated poor. Birkenhauer et al. replaced 38 Spiron artificial hips for 34 patients, and the preoperative Harris hip score was 51 points (24–76). After one year of surgery, more than 20 patients were examined, and the Harris hip score increased to 94 points (86–100) [16]. Lugeder et al. replaced 28 Spiron hips in 26 patients. The Harris hip score before surgery was 55.4 points; after three months of testing, it was 90.5 [22]. Logroscino et al. conducted a retrospective case-control study to compare the results between 46 patients using short stems and 50 patients using traditional stems with a mean follow-up of 30 months. In the postoperative course, the short stems group seems to have better results in terms of the Harris hip score (93 vs. 91.7; *p*=0.18) [23].

According to Banerjee et al., the rate of fractures around the short shaft in general is 1.4% (0–7%), and the rate for the Mayo shaft is 4.2% (0%–7%), lateral flare design is 2.4% (1.1%–5.7%), and shortened proximally coated is 0.8% (0–2.1%) [21]. Ishaque et al. reported a case of a 61-year-old patient suffering a fracture of the implant four years after primary implantation of an Cut short-stemmed prosthesis [24]. Our study did not encounter any cases of articular shaft fractures or bone fractures around the shaft. We met 1 case (accounting for 0.8%) of acetabulum fracture during surgery. We had to screw three screws to fix the acetabulum and give the patient late compression. After checking, the bone healed well, and the acetabulum did not move.

According to Banerjee S., the average rate of thigh pain after short-stem hip replacement is 0.4% (0–2.7%). Also, according to the author, the thigh pain rate is less than 3% for short stem [21], much higher than the traditional-stem joint's 10–20% thigh pain rate. Some new short-stem designs, such as the extended lateral flare fit joint or the femoral neck thread femoral stem, can eliminate the complication of thigh pain [25]. During an average follow-up period of 67 months, we encountered 11 cases (accounting for 9.2%) with persistent symptoms of discomfort in the greater trochanter area but not frequently and not to the point of thigh pain.

The phenomenon of femoral stem subsidence and femoral stem displacement is very noticeable for short-stem joints [26]. In 2013, according to a study by Banerjee S., the short-shaft joint displacement rate was 1.4% (0–7). The stress shielding phenomenon accounts for 5%; this rate for Mayo rolling is 5.6% (4%–6%) [21]. Stulberg and Patel encountered 1 Case (1.4%) of joint subsidence after three months [12]. Kim et al., in a study of the Fitmore short-stem joint, encountered 18/72 patients (25%) with 1.5 mm subsidence in two years. However, the authors said that no subsequent modifications were required [27]. According to Krismer et al., longitudinal subsidence over 1.5 mm predicts a high risk of failure in joint replacement [28]. We encountered 12 cases (10.1%) of bone loss around the femoral stem and 12 cases (10.1%) of femoral stem displacement, all of which were angulation displacements.

Limb discrepancy is the most common complaint after hip replacement [29]. After surgery, if the lower limb deviation is 5 mm, it is classified as having equal leg length [30]. The leading cause of limb imbalance is the joint not being in the correct position. Edeen et al. encountered 32% of the patients complaining of inequality in the lower limbs after joint replacement [31]. In the study of Ettinger et al., the difference in length of the two limbs was 2.8 mm for the Minihip joint and 3.6 mm for the Metha short-stem joint [32]. Edwards et al. suggested that if the difference between the two limbs is 10 mm or more after hip replacement, it will affect gait, cause pain, and quickly erode the acetabular lining [33]. In our study, the average leg length discrepancy between the two limbs before surgery was 9.5 ± 2.3 mm and 24 months after surgery was 3.4 ± 2.9 mm, which is intensely significant.

Innovations play an essential role in the success of orthopedic surgery. However, creating a change in clinical practice requires the consensus of many experts in the field based on strong evidence from research. Going back in history, we can see significant changes in the THA process, such as the emergence of dual modular stems, also known as bimodal stems, in 1987. Although modular systems dual therapy was initially touted as offering some advantages, the evidence supporting these benefits still needs to be discovered [34]. Similarly, another example of an innovation trap mentioned in the literature is the Profemur Z fully-modular femoral stem [35]. Although short-stem implants have shown promising results in clinical practice, further studies with larger sample sizes, more diverse pathologies (not simply aseptic necrosis of the femoral head), and randomized controlled clinical trials are still needed to evaluate possible patient complications and conduct further subgroup analyzes to find patient groups that truly benefit from this surgical method.

## 5. Conclusion

The Spir on short stem has certain advantages suitable for young patients, restoring the hip anatomy and significantly improving postoperative hip function. However, this is a complex technique that needs to be performed by experienced surgeons who have practiced for a long time. We encourage using the short-stem implant during primary total hip arthroplasty in Vietnamese adult patients [36, 37].

## Figures and Tables

**Table 1:** Characteristics of study subjects.

Patient characteristics	*N* (%)
Age	
The average age	41.22 ± 5.97
25–30	3 (3.3%)
31–40	36 (39.6%)
41–50	52 (57.1%)
Gender	
Male	89 (97.8%)
Female	2 (2.2%)
Disease stage	
IV	49 (41.2%)
V	60 (50.4%)
VI	10 (8.4%)
Surgical side	
Right	23 (25.3%)
Left	40 (44%)
Both	28 (30.7%)

**Table 2:** Characteristics of implant.

Implant characteristics	*N* (%)
Acetabulum (mm)	
46	9 (7.6%)
48	43 (36.1%)
50	59 (49.6%)
52	8 (6.7%)
Joint head diameter (mm)	
−5 (S: small)	95 (79.8%)
+0 (M: medium)	20 (16.8%)
+5 (L: large)	4 (3.4%)
Femoral stem diameter (mm)	
18	10 (8.4%)
20	73 (61.3%)
22	36 (30.3%)
Femoral stem length (mm)	
45	40 (33.6%)
50	64 (53.8%)
55	12 (10.1%)
60	3 (2.5%)

**Table 3:** Surgical results.

Patient characteristics	*N* (%)	*p*
Harris hip scores		
Before surgery	45.56 ± 3.22	<0.001
24 months after surgery	92.00 ± 4.47	
Classification of results after 24 months of surgery according to Harris		
Very good	94 (79%)	
Good	22 (18.5%)	
Medium	3 (2.5%)	
Poor	0	
Difference in length of 2 legs (mm)		
Before surgery	9.5 ± 2.3	0.038
24 months after surgery	3.4 ± 2.9	

## Data Availability

The datasets used and/or analyzed during the current study are available from the corresponding author on reasonable request.
